# Surgery for subacromial impingement syndrome and occupational biomechanical risk factors in a 16-year prospective study among male construction workers

**DOI:** 10.5271/sjweh.4075

**Published:** 2023-02-27

**Authors:** Charlotte Lewis, Jens Wahlström, Sebastian Mukka, Per Liv, Bengt Järvholm, Jennie A Jackson

**Affiliations:** 1 Department of Public Health and Clinical Medicine, Umeå University, Umeå, Sweden; 2 Department of Surgical and Perioperative Sciences (Orthopaedics), Umeaå University, Umeå, Sweden; 3 Department of Occupational Health Sciences and Psychology, University of Gävle, Gävle, Sweden

**Keywords:** ergonomics, grip force, hand-arm vibration, hand-held tool, posture, shoulder, static posture, upper-arm load

## Abstract

**Objective:**

The aim of this study was to assess the association between occupational biomechanical exposures and the occurrence of surgical treatment for subacromial impingement syndrome (SIS).

**Methods:**

A cohort of 220 295 male constructions workers who participated in a national occupational health surveillance program (1971–1993) were examined prospectively over a 16-year follow-up period (2001–2016) for surgically treated SIS. Worker job title, smoking status, height, weight, and age were registered on health examination. Job titles were mapped to 21 occupational groups based on tasks and training. A job exposure matrix (JEM) was developed with exposure estimates for each occupational group. Surgical cases were determined through linkage with the Swedish national in- and outpatient registers. Poisson regression was used to assess the relative risks (RR) for each biomechanical exposure.

**Results:**

The total incidence rate of surgically treated SIS over the 16-year observation period was 201.1 cases per 100 000 person-years. Increased risk was evident for workers exposed to upper-extremity loading (push/pull/lift) (RR 1.45–2.30), high hand grip force (RR 1.47–2.23), using handheld tools (RR 1.52–2.09), frequent work with hands above shoulders (RR 1.62–2.11), static work (RR 1.77–2.26), and hand-arm vibration (RR 1.78–2.13). There was an increased risk for SIS surgery for all occupational groups (construction trades) compared with white-collar workers (RR 1.56–2.61).

**Conclusions:**

Occupational upper-extremity load and posture exposures were associated with increased risk for surgical treatment of SIS, which underlines the need for reducing workplace exposures and early symptom detection in highly exposed occupational groups.

Shoulder disorders are common in the general population, with annual prevalence rates of 5–48% depending on case definition and population (1, 2). Subacromial impingement syndrome (SIS) is a common diagnosis that comprises multiple disorders with overlapping symptoms (3). Two etiologies have been suggested for SIS onset. The first, *intrinsic impingement*, suggests tendon degeneration from overuse, tension overload, or trauma (3). The second, *extrinsic impingement*, suggests inflammation and tendon degeneration as a result of external mechanical compression, for example, from structural narrowing of the subacromial space leading to compression of the supraspinatus tendon, subacromial bursa, and/or the long head of the biceps brachii tendon (3).

Primary treatment of SIS is non-operative, and includes rest during the acute phase combined, if necessary, with non-steroidal anti-inflammatory drugs (NSAID), followed by exercise focused on improving rotator cuff and scapular stabilizer muscle function (4). For symptomatic individuals for whom conservative treatments prove ineffective, surgical treatment is a subsequent alternative (5).

Individual risk factors for shoulder pain include being female, obese, of an older age, having certain coexisting medical disorders and smoking (6, 7). Occupational groups with repetitive shoulder movements, upper-arm elevation and arm/shoulder load have been associated with a higher risk for shoulder pain including construction tradesmen (6, 8–11). Within this sector, exposure to heavy upper-extremity loading, often in awkward postures and work with arms above shoulder height is common, as is use of vibrating hand tools (12–14).

The aim of the current study was to assess the association between occupational biomechanical exposure and the occurrence of SIS in a large cohort of construction workers.

## Methods

### Study design and population

In this cohort study, we assessed the association between occupational biomechanical postural and load exposures and surgical treatment for SIS. The Regional Ethical Review Board in Umeå approved the study (2017/16-31).

The cohort comprised 389 132 Swedish construction workers who participated in health examinations between 1971 and January 1993. While participation was voluntary, ≥80% of eligible workers completed at least one health examination, and the average number of health examination was three. Amongst others height, weight, age, smoking status, and specific trade (‘job title’) were recorded on examination. Only male construction workers were included in the analysis as the percentages of women in many occupational groups were low. Women constituted approximately 5% of the total cohort. The follow-up period was 2001–2016.

**Figure 1:**
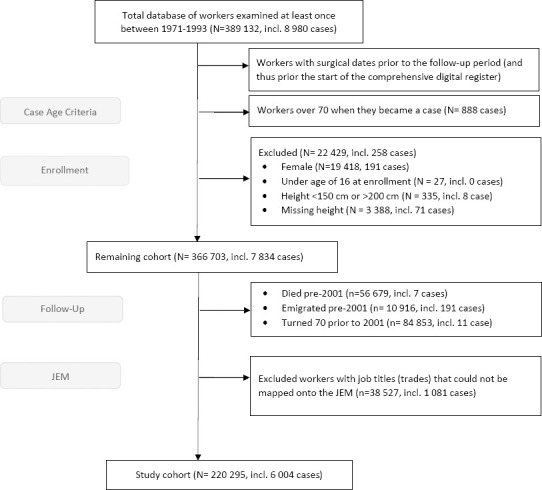
Flow diagram outlining exclusions from total construction worker cohort and resulting study cohort.

Workers were also excluded who: were <16 years at their first health examination; were unusually short (<150 cm) or tall (>200 cm); had missing record of height; and had died or emigrated or turned 70 years of age before 2001. The older workers were excluded due to the unlikelihood of being active in the industry at this age and because surgical treatment for SIS at older ages may result from diseases, common among older workers but unrelated to occupational exposure. Data on emigration and deaths were obtained from the Total Population Register, held by Statistics Sweden. In addition, workers for whom no job title was recorded at any of the medical examinations or who were classified in the non-specific (‘other’) work group were excluded since they could not be mapped onto the job exposure matrix (JEM) (table 1). The remaining 220 835 workers comprised the study cohort (figure 1).

**Table 1:** Biomechanical risk factors included in the job exposure matrix. On average, over a working day, and across the sub-jobs included in the occupational group.

Exposure	Rating
Frequency of working with hands above shoulder height	1–3 ^a^
Magnitude of upper extremity loading (can result from push/pull/lift)	1–3 ^a^
Frequency of high grip force	1–3 ^a^
Frequency of handheld tool use	1–3 ^a^
Frequency of using a handheld tool in a fixed position	1–3 ^a^
Frequency of upper extremity static work	1–3 ^a^
Magnitude of hand-arm vibration (HAV)	1–3 ^b^

a 1 = low, 2 = moderate, 3 = high

b 1 = none, 2 = acceptable, 3 = high

### Case definition

SIS cases were defined using both main diagnosis and surgical treatment procedures. Cases comprised individuals with a main diagnosis according to the 10^th^ International Classification of Diseases of: M75.1 rotator cuff syndrome, M75.2 bicipital tendinitis, M75.3 calcific tendinitis, M75.4 impingement, or M75.5 bursitis paired with at least one of the following surgical codes: NBA diagnostic procedure, NBE surgery on ligament, NBF surgery of synovia, NBG reconstruction & arthrodesis, NBH dislocation and loose bodies, NBK surgery on bone, NBL surgery on muscle and tendon, or NBM surgery on fascia, tendon synovial sheath, bursae. All diagnosis and surgical code data came from the Swedish national inpatient and outpatient registries, and only first-time surgeries (within the follow-up period) were considered. Data were obtained from 2001, the start of the period for which computerized data were available for both registries, until 2016. Register linkage was achieved using the unique personal number assigned to each Swedish resident with computerized in- and outpatient data and from 2001 until the end of 2016. For the distribution of the diagnostic codes, see the supplementary material (www.sjweh.fi/article/4075) table S1.

### Individual factors

Self-reported smoking status was extracted from the same health examination that provided job title (the last examination), and workers were classified into never, ever, and unknown categories. Height and weight were taken from the first health examination; workers were classified by body mass index (BMI) into underweight (<18.5 kg/m^2^), normal (18.5–24.9 kg/m^2^), overweight (25–29.9 kg/m^2^) and obese (≥30 kg/m^2^) groups.

### Biomechanical exposure

For each worker, job title was extracted from the last health examination record (1971–1993). Job titles were coded using occupational work codes applied in the Swedish construction industry at the time of the health exam. Prior to 1986, 212 individual job title codes were used, while 90 job title codes were used from 1986 onwards. The job titles were mapped to 21 occupational groups developed by technical experts of the industry to group workers performing similar work tasks and having similar training.

A job exposure matrix (JEM) was developed to categorize full-day biomechanical exposure in each occupational group. We chose seven exposures deemed relevant by the available literature on shoulder disorders to be included in our JEM (see table 1 for a summary of exposure). Two experts independently rated the average exposure intensity or frequency over a working day assisted by ergonomic assessments conducted in the 1970s and job descriptions for each job title. All ratings were done blinded to the number of SIS surgery cases in each occupational group. Ratings were compared and discussed by the experts to reach consensus. A single expert performed the vibration ratings for each occupational group. Ratings were made for all occupational groups except for ‘other work’ (supplementary table S2). Exposure estimates were assigned to individuals based on the JEM ratings for their occupational group.

### Self-reports and pain ratings

Self-reported estimates of occupational exposure and ratings of pain experienced during the previous 12 months were collected at health examinations conducted between 1989 and 1993, including 69 767 of the men in the cohort (32%). The workers responded to each question using a 5-point scale to grade frequency of working with hands above shoulders and severity of pain in the shoulders. Workers who subjectively rated never or rarely having shoulder pain were grouped to represent a healthy population sub-group, which was used as the reference group in an additional set of analyses.

### Statistical analysis

Person-years were calculated from 2001 until year of SIS surgery, or end of the observation period (31 December 2016), censoring for death, emigration and becoming 70 years of age. Follow-up was split into four time periods, each of four years, to account for differences in incidence across both calendar time and increasing age of workers. Poisson regression was used to estimate relative risks (RR), incidence rate ratios (IRR), and 95% confidence intervals (CI) for surgery, adjusted for age, BMI, smoking status, height, and time periods. Age, BMI, and height were adjusted for using restricted cubic splines with three knots placed at the 10^th^, 50^th^ and 90^th^ percentiles of corresponding variable distribution. Crude relative risks are presented in supplementary table S3.

White-collar workers from the same construction cohort were used as reference in the analysis of biomechanical exposure and the comparisons between occupational groups. RR for surgical treatment for SIS were also calculated on the subset of workers who had subjectively rated never or rarely having shoulder pain (healthy sub-group) in order to analyze the relative risks on subjects without pre-existing shoulder pain. Due to low numbers of cases in the white-collar workers in this subset analysis, occupational groups rated as having low exposure in our JEM formed the reference group. Additional analyses were also performed for a subset of workers still in construction trade at least one year during a five-year period prior to our follow-up period in order to obtain workers for whom the JEM scores more accurately predicted their recent occupational exposure. Employment status was determined through a linkage of the study database with Statistics Sweden’s LISA register (longitudinal integrated database for health insurance and labor market studies).

## Results

The study cohort (N=220 295) accumulated at total number of 2 985 269 person-years of observation. There were in total 6 004 SIS surgery cases included in the analysis. The 16-year IRR was 201.1 cases per 100 000 person-years.

The risk of surgical treatment for SIS increased with age up to the 55–64 year old category (table 2). Compared with subjects in the height category 170–180 cm, shorter workers were at increased risk for SIS surgery, while subjects >180 cm had a decreased risk for SIS surgery. Smoking increased the risk of surgical treatment of SIS with a RR of 1.33 (95% CI 1.26–1.40) for ever- compared to never smokers. Being overweight or obese increased the risk of surgical treatment of SIS with an RR of 1.13 (95% CI 1.06–1.20) and 1.33 (95% CI 1.16–1.52), respectively (table 2).

**Table 2:** Incidence rate ratios (IRR) per 100 000 person-years and relative risks (RR) according to age, height, smoking habits and body mass index (BMI). RR are presented as crude estimates, from unadjusted models. [CI=confidence interval]

	N	Person-years	Cases	IRR	RR	95% CI
Age (years)						
25–34	27 413	147 119	39	27	1.00	
35–44	81 734	640 479	670	105	2.99	2.16–4.13
45–54	130 844	919 132	1929	210	5.54	4.03–7.62
55–64	130 785	991 617	2811	284	7.26	5.29–9.98
≥65	84 964	286 922	555	193	4.66	3.36–6.46
Height (cm)						
150–160	468	5500	17	309	1.47	0.91–2.37
160–170	21 966	272 714	630	231	1.10	1.01–1.20
170–180	115 575	1 537 559	3224	210	1.00	
180–190	75 312	1 065 939	1968	185	0.88	0.83–0.93
190–200	6 974	103 557	165	159	0.76	0.65–0.89
Smoking						
Never	98 843	1 408 797	2437	173	1.00	
Ever	110 421	1 432 620	3299	230	1.33	1.26–1.40
Unknown	11 031	143 852	268	186	1.08	0.95–1.22
BMI						
Underweight	3 580	54 314	85	156	0.81	0.65–0.99
Normal weight	153 801	2 151 781	4178	194	1.00	
Overweight	55 463	688 646	1507	219	1.13	1.06–1.20
Obese	7226	87 274	225	258	1.33	1.16–1.52

All included upper-extremity loads and postures were associated with increased risk of SIS surgery and a dose–response pattern was suggested for upper-extremity loading, and frequency of high grip force (table 3). The highest RR were for workers with high exposure to upper-extremity loading (RR 2.30, 95% CI 1.88–2.81), high frequency of high grip force (RR 2.23, 95% CI 1.82–2.73) and moderate exposure of static work (RR 2.26, 95% CI 1.84–2.77). All occupational groups had higher RR for SIS surgery than the reference white-collar workers group (table 4). Among the construction trades, risk for SIS surgery was highest for brick layers, floor layers, sheet-metal workers, wood workers, glass workers and repairers (RR 2.16–2.61).

**Table 3:** Biomechanical risk factors and the incidence rate ratios (IRR) per 100 000 person-years and relative risk (RR) for shoulder impingement syndrome (SIS) surgery (N=220 295) in the study cohort of construction workers. RR were adjusted for BMI, smoking, age, and calendar time. The white-collar workers were used as reference. [CI=confidence interval]

	N	Person-years	Cases	IRR	RR	95% CI
Frequency of working with hands above shoulder height						
Reference	7491	89 855	99	110	1.00	
Low	51 075	658 207	1165	177	1.62	1.32–2.00
Moderate	143 721	1 986 815	4248	214	2.11	1.72–2.58
High	18 008	250 392	492	196	1.97	1.58–2.44
Magnitude of upper extremity loading (push/pull/lift)						
Reference	7491	89 855	99	110	1.00	
Low	33 729	422 996	680	161	1.45	1.17–1.79
Moderate	95 040	1 317 089	2525	192	1.90	1.55–2.33
High	84 035	1 155 329	2700	234	2.30	1.88–2.81
Frequency of high grip force						
Reference	7491	89 855	99	110	1.00	
Low	36 742	462 297	753	163	1.47	1.19–1.81
Moderate	73 639	1 029 742	1 956	190	1.90	1.55–2.33
High	102 423	1 403 375	3 196	228	2.23	1.82–2.73
Frequency of hand-held tool use						
Reference	7491	89 855	99	110	1.00	
Low	38 970	493 157	831	169	1.52	1.24–1.88
Moderate	17 364	236 790	512	216	2.08	1.68–2.58
High	156 470	2 165 467	4562	211	2.09	1.71–2.55
Frequency of using a handheld tool in a fixed position						
Reference	7491	89 855	99	110	1.00	
Low	72 329	94 6628	1756	186	1.74	1.42–2.14
Moderate	9551	132 139	285	216	2.09	1.66–2.63
High	130 924	1 816 647	3864	213	2.09	1.71–2.56
Frequency of upper extremity static work						
Reference	7491	89 855	99	110	1.00	
Low	62 098	824 882	1531	186	1.79	1.46–2.19
Moderate	86 243	1 188 882	2755	232	2.26	1.84–2.77
High	64 463	881 650	1619	184	1.77	1.44–2.17
Magnitude of hand-arm vibration (HAV)						
Reference	7491	89 855	99	110	1.00	
Low	90 717	1 194 674	2252	189	1.78	1.45–2.18
Moderate	98 057	1 396 951	2995	214	2.13	1.74–2.61
High	24 030	303 789	658	217	2.04	1.65–2.52

**Table 4:** Incidence rate ratios (IRR) per 100 000 person-years and relative risks (RR) for SIS surgery, adjusted for body mass index, smoking, age, and calendar time according to occupational group. Occupational groups shown in order of ascending RR. [CI=confidence interval]

	N	Person-years	Cases	IRR	RR	95% CI
White-collar workers	7491	89 855	99	110	1.00	
Foremen	20 191	254 641	352	138	1.28	1.02–1.60
Drivers	3014	37 567	69	184	1.56	1.15–2.13
Refrigerator technicians	1071	15 604	26	167	1.62	1.05–2.50
Heavy machinery operators	8209	104 846	199	190	1.65	1.29–2.10
Asphalt workers	3013	39 301	73	186	1.69	1.25–2.29
Electricians	30 729	444 132	746	168	1.72	1.39–2.12
Insulators	2143	30 005	55	183	1.79	1.28–2.49
Roofers	1071	14 523	28	193	1.81	1.19–2.76
Plumbers	18 388	248 046	496	200	1.94	1.56–2.42
Painters	18 008	250 392	492	196	1.98	1.59–2.46
Concrete workers	20 263	257 274	536	208	2.00	1.61–2.48
Rock workers	1808	21 117	49	232	2.02	1.43–2.85
Crane operators	2315	25 942	60	231	2.02	1.46–2.78
Preparatory workers	7813	104 651	227	217	2.06	1.63–2.62
Brick layers	6467	83 905	178	212	2.16	1.69–2.77
Floor layers	4378	61 132	131	214	2.17	1.67–2.82
Sheet-metal workers	9858	140 683	327	232	2.30	1.83–2.88
Wood workers	49 878	705 395	1710	242	2.42	1.97–2.96
Glass workers	2228	30 860	78	253	2.47	1.83–3.33
Repairers	1959	25 398	73	287	2.61	1.93–3.54

In the analysis restricted to the healthy sub-group (workers reporting no shoulder symptoms on examination, 1989–1993) the associations between exposure and SIS surgery showed similar associations (supplementary table S4).

## Discussion

Increased risk was evident for workers exposed to upper-extremity loading (push/pull/lift), high hand grip force, handheld tool uses, frequent work with hands above shoulders, static work, and hand-arm vibration (RR 1.45–2.30). Most of the exposure risk factors suggested exposure–response associations. All occupational groups had an increased risk for surgically treated SIS compared to white-collar workers, with highest risk shown for brick layers, floor layers, sheet-metal workers, wood workers, glass workers and repairers. Age, short stature, smoking and BMI were also identified as risk factors.

For exposure estimates, we used a JEM based on historical records comprising detailed descriptions of biomechanical exposures and tasks performed within each job title. This minimizes the risk of recall bias and is often considered the best available method for retrospective exposure assessment in cohort studies (15). By having the historical description of the actual included occupations, we minimize possible discrepancies due to contextual differences (time, geographical) in our exposure assessment. However, the experts’ ratings were not validated against technical measurement. Also, we assigned exposures at the group level and cannot therefore account for individual differences in work strategies or specific tasks performed within a job title. Lastly, exposure within an occupational group can change over time, due to changing work processes and work technique. The exposure assessment classified in our JEM can be seen as an average exposure over the studied time period for each occupational group, since it did not take these changes into account.

Many of the biomechanical factors in the JEM are correlated (supplementary table S5) and for some exposures it is difficult to draw conclusions regarding specific underlying pathomechanistic pathways. For example, people working with hand-held tools likely also have vibration exposure and possibly high grip force and vice versa. Also, the JEM created for this study did not focus on the cumulative aspects of different biomechanical exposures.

In the total study cohort, no exclusion was made based on existing shoulder symptoms since a symptomatic period (often lasting months or years) usually precedes becoming a surgical case. Having shoulder symptoms could have motivated workers to change jobs (trades) to minimize occupational shoulder exposures, which would have led to an under estimation of risk. An analysis on a subset of workers still in construction trade at least one year during a five-year period prior to our follow-up period also showed similar results in risk estimates; this argues against a healthy survivor effect (supplementary table S6). In the analysis restricted to the healthy sub-group (supplementary table S4) which comprised only workers who had no shoulder symptoms at inclusion, risk factor findings were similar to the total study cohort. The consistent findings support the evidence that occupational exposure increased the risk for SIS surgery. Since the reference groups in the healthy cohort analyses were different, the RR could not be compared with the total study cohort.

Data from a Danish national cohort presented an incidence of 106 cases of first-time surgical treatment of SIS per 100 000 person-years (16), compared to our 201.1 cases per 100 000 person-years. The higher IRR in the present study cohort is to be expected since it is based on trades with high physical exposure as opposed to the general population in the Danish study.

Our results confirm findings from previous studies reporting exposure to upper-extremity loading being a risk factor for SIS surgery as well for shoulder complaints (6, 10, 17). A study by Dalbøge et al (17) investigated exposure–response with regards to cumulative exposure rather than the amplitude of the exposure and saw an exposure-response pattern between force and SIS surgery. Exposure to vibration was associated to SIS surgery in the present study, which is confirmed in other studies not adjusting for biomechanical factors (16, 17), while no association is found when adjusting for biomechanical factors (18). In our study, there was a high correlation between most biomechanical factors and HAV exposure, which prevented from controlling for the correlates in the same model. In practice, it is hard to separate the effect of the two risks since exposure to HAV is always accompanied by an exposure to hand force since it is required to operate hand-held tools. However, a study by Bovenzi et al (19) suggested that exposure to both biomechanical factors and HAV contributed in a multiplicative way to the occurrence of musculoskeletal disorders in female workers. Regarding factors relating to upper-extremity postures, such as working with hands above shoulders, associations have been presented with increased risk for SIS surgery (8, 16, 17) as well for shoulder complaints (6, 20). We found that short stature was a risk factor for SIS surgery, which has not previously been addressed in similar studies (8, 17). One plausible explanation is that shorter stature workers may be more prone to work with elevated arms, which we also identified as a risk factor. This highlights the importance of being able to adjust the physical work environment to fit workers of various sizes. We also found smoking increased the risk for SIS surgery, which is in agreement with findings by Dalbøge et al (17). Our results also confirm several previous studies which showed increased risk of musculoskeletal disorders with increased age (21, 22). This increase can be an effect of ageing, per se, and/or due to cumulative exposures to biomechanical risk factors. Primary and secondary preventative measures to minimize cumulative exposures and disease progression are recommended, and consideration of altered work tasks for aging workers may be warranted.

The present study included a large cohort, detailed assessment of biomechanical exposures, prospective follow-up and a clear case criterion. Associations between high biomechanical exposure and risk of surgical treatment for SIS can reflect both risk factors for developing SIS, but also be caused by physicians admitting subjects with physically demanding job’s (ie, a high biomechanical load). Misclassification in the registers would be independent of the exposure variables and therefore would tend towards decreased relative risk estimates.

The reference group consisted of white-collar workers from the same cohort. These workers were selected as they, in general, have no heavy physical exposure at work even though they are employed within the same organization. Though access to healthcare in some countries is strongly related to socioeconomic status, this is not the case in Sweden due to socialized health care, hence it should not have impacted the access to surgery in our population. Socioeconomic status is related to lifestyle factors such as overweight and smoking, which we controlled for in the analyses. However, we cannot rule out that some socioeconomical differences existed between construction trade and white-collar workers.

The indications for surgical treatment of SIS have been debated and have varied over time (23, 24). We adjusted for calendar time in our analysis in order to minimize a possible time bias, though some variation could have occurred geographically due to differences in availability of surgery. However, we do not expect that the likelihood of admission would be related to any of the individual or biomechanical exposure variables. All construction workers within the cohort had good access medical healthcare, which is nearly free of charge in Sweden.

Exposures in the construction trades can differ between countries, due to variations in material use, work organization and working technique. Therefore, the RR within the different occupational groups might not be directly transferrable outside of Sweden.

SIS etiology has been postulated from both intrinsic (intratendinous) and extrinsic (extratendinous) factors (25). Our findings of both postural and force related exposure factors leading to increased risk are in agreement with previously suggested intrinsic pathways. For example, intrinsic factors can include pathological changes in supraspinatus as a result of muscle weakness due to tension overload due to excessive work with elevated arms, overuse with repetitive microtrauma, for example, overhead hammering or handling of a vibrating tool, or degenerative tendinopathy with tendon tears. Work in an overhead position can result in eccentric contraction of the supraspinatus leading to muscle overload. Palmerud et al (26) showed an increased pressure in rotator cuff muscles during arm elevation as well as during hand load which could be an additional pathomechanism for developing SIS. Repetitive microtrauma and eccentric contraction also facilitate tendinous changes which might contribute to a higher incidence of SIS. An altered adductor co-contraction pattern during abduction has been proposed to perpetuate symptoms in SIS, resulting in a reduction of caudally directed forces on the humeral side of the joint which may lead to a repetitive overloading of the subacromial tissues (26).

### Implications

This study further supports that occupational upper-extremity loads and postures with elevated arms are associated with increased risk for surgical treatment for subacromial impingement syndrome. It highlights the need for monitoring and reducing workplace exposure, as well as detecting early signs of shoulder symptoms in highly exposed occupational groups. Reducing mechanical loading is in line with the broader ergonomic literature on preventing work-related upper-extremity disorders.

## Supplementary material

Supplementary material
