# Supplementary material for: Surgery for subacromial impingement syndrome and occupational biomechanical risk factors in a 16-year prospective study among male construction workers
Source: Scand J Work Environ Health. 2023 Feb 27;49(2):156–63. doi: 10.5271/sjweh.4075 (PMC10577012; doi:10.5271/sjweh.4075)
Supplement: Supplementary material [file SJWEH-49-156-S001.pdf]

Surgery for subacromial impingement syndrome and occupational biomechanical risk factors in a 16-year prospective study among male construction workers[1]

Charlotte Lewis, PhD,[2] J Wahlström, PhD S Mukka, PhD P Liv, PhD Bengt Järvholm, PhD, JA Jackson, PhD

1. Supplementary material
2. Correspondence to: Charlotte Lewis, Department of Public Health and Clinical Medicine, Sustainable health, SE-901 87 Umeå University, Sweden. [E-mail: charlotte.lewis@regionvasterbotten.se]

Table S1. Distribution of diagnostic codes (10th International Classification of Diseases), of included cases.

Diagnose code		N (%)
M75.1	rotator cuff syndrome	2 502 (41.7)
M75.2	bicipital tendinitis	100 (1.7)
M75.3	calcific tendinitis	113 (1.9)
M75.4	impingement	3 209 (53.4)
M75.5	bursitis	80 (1.3)

Table S2. JEM mapping for all included occupational groups.

	Frequency of working with hands above shoulder height [a]	*Magnitude of upper extremity loading [a]*	*Frequency of high grip force [a]*	*Frequency of handheld tool use [a]*	*Frequency of upper extremity static work [a]*	*Frequency of using a handheld tool in a fixed position [a]*	*Magnitude of hand-arm vibration (HAV) [b]*
Asphalt workers (1)	1	2	1	1	1	1	1
Rock workers (2)	2	3	3	3	3	3	3
Concrete workers (3)	2	3	3	3	2	3	3
Wood workers (4)	2	3	3	3	2	3	2
Brick layers (5)	2	2	2	3	1	1	1
Floor layers (6)	1	2	2	2	1	2	2
Heavy Machinery operators (7)	1	1	1	1	3	1	1
Crane operators (8)	1	1	1	1	3	1	1
Drivers (9)	1	1	1	1	3	1	1
Glass workers (10)	2	3	3	1	1	1	1
Insulators (11)	2	2	2	2	2	2	2
Refrigerator technicians (12)	1	2	2	2	2	2	2
Plumbers (13)	2	2	3	3	3	3	1
Painters (14)	3	2	2	3	1	1	1
Sheet-metal workers (15)	2	3	3	3	2	3	2
Electricians (16)	2	2	2	3	3	3	2
Foremen (17)	1	1	1	1	1	1	1
White Collar workers (18)	1	1	1	1	2	1	1
Repairers (19)	2	2	2	2	2	2	3
Preparatory workers (20)	1	2	2	2	1	1	1
Roofers (21)	1	2	2	3	2	1	1

[a] 1 = low, 2 = moderate, 3 = high
[b] 1 = none, 2 = acceptable, 3 = high

Table S3. Biomechanical risk factors and the incidence rate (IR) and relative risk (RR) for SIS surgery (n=220 295) in the study cohort of construction workers. RR are presented as crude estimates, from unadjusted models. The white-collar workers were used as reference.

	N	person-years	cases	IR	RR	95% CI
Frequency of working with hands above shoulder height						
reference	7 491	89 855	99	110	1.00	-
low	51 075	658 207	1 165	177	1.61	1.31-1.97
moderate	143 721	1 986 815	4 248	214	1.94	1.59-2.37
high	18 008	250 392	492	196	1.78	1.44-2.21
Magnitude of upper extremity loading						
reference	7 491	89 855	99	110	1.00	-
low	33 729	422 996	680	161	1.46	1.18-1.80
moderate	95 040	1 317 089	2 525	192	1.74	1.42-2.13
high	84 035	1 155 329	2 700	234	2.12	1.74-2.59
Frequency of high grip force						
reference	7 491	89 855	99	110	1.00	-
low	36 742	462 297	753	163	1.48	1.20-1.82
moderate	73 639	1 029 742	1956	190	1.72	1.41-2.11
high	102 423	1 403 375	3196	228	2.07	1.69-2.52
Frequency of handheld tool use						
reference	7 491	89 855	99	110	1.00	-
low	38 970	493 157	831	169	1.53	1.24-1.88
moderate	17 364	236 790	512	216	1.96	1.58-2.43
high	156 470	2 165 467	4 562	211	1.91	1.57-2.33
Frequency of using a handheld tool in a fixed position						
reference	7 491	89 855	99	110	1.00	-
low	72 329	946 628	1 756	186	1.68	1.38-2.06
moderate	9 551	132 139	285	216	1.96	1.56-2.46
high	130 924	1 816 647	3 864	213	1.93	1.58-2.36
Frequency of upper extremity static work						
reference	7 491	89 855	99	110	1.00	-
low	62 098	824 882	1 531	186	1.68	1.37-2.06
moderate	86 243	1 188 882	2 755	232	2.10	1.72-2.57
high	64 463	881 650	1 619	184	1.67	1.36-2.04
Magnitude of hand-arm vibration (HAV)						
reference	7 491	89 855	99	110	1.00	-
low	90 717	1 194 674	2 252	189	1.71	1.40-2.09
moderate	98 057	1 396 951	2 995	214	1.95	1.59-2.38
high	24 030	303 789	658	217	1.97	1.59-2.43

N – number workers; IR – incidence rate per 100 000 person-years; RR – relative risk; CI – confidence interval

Table S4. Biomechanical exposure and the incidence rate (IR) and relative risk (RR) for SIS surgery (n=33 669) in the healthy subgroup. RR were adjusted for BMI, smoking, age, and calendar time. The lowest exposure category was used as reference.

	N	person-years	cases	IR	RR	95% CI
Frequency of working with hands above shoulder height						
low	8 931	123 922	123	99	1.00	-
moderate	22 391	330 398	467	141	1.60	1.30-1.95
high	2 347	34 399	51	148	1.78	1.28-2.48
Magnitude of upper extremity loading						
low	5 951	80 482	73	91	1.00	-
moderate	14 527	213 539	268	126	1.60	1.23-2.08
high	13 191	194 698	300	154	1.95	1.50-2.52
Frequency of high grip force						
low	6 452	87 507	83	95	1.00	-
moderate	11 221	165 833	203	122	1.51	1.17-1.96
high	15 996	235 379	355	151	1.82	1.42-2.32
Frequency of handheld tool use						
low	6 777	92 355	90	97	1.00	-
moderate	2 858	41 792	52	124	1.39	0.98-1.96
high	24 034	354 572	499	141	1.66	1.32-2.09
Frequency of using a handheld tool in a fixed position						
low	11 591	162 298	182	112	1.00	-
moderate	1 473	21 511	32	149	1.38	0.95-2.02
high	20 605	304 910	427	140	1.34	1.13-1.60
Frequency of upper extremity static work						
low	9 573	135 767	164	121	1.00	-
moderate	14 642	214 428	318	148	1.25	1.03-1.50
high	9 454	138 524	159	115	0.96	0.77-1.19
Magnitude of hand-arm vibration (HAV)						
low	14 396	202 979	237	117	1.00	-
moderate	16 138	242 049	340	140	1.30	1.10-1.54
high	3 135	43 691	64	146	1.25	0.95-1.65

N – number workers; IR – incidence rate per 100 000 person-years; RR – relative risk; CI – confidence interval

Table S5. Spearman correlation coefficients between pairs of biomechanical exposures.

	Frequency of working with hands above shoulder height	*Magnitude of upper extremity loading*	*Frequency of high grip force*	*Frequency of handheld tool use*	*Frequency of upper extremity static work*	*Frequency of using a handheld tool in a fixed position*	*Magnitude of hand-arm vibration (HAV)*
Frequency of working with hands above shoulder height	1.0	0.5	0.6	0.9	0.1	0.4	0.3
Magnitude of upper extremity loading		1.0	0.9	0.7	0.0	0.7	0.7
Frequency of high grip force			1.0	0.8	0.2	0.8	0.6
Frequency of handheld tool use				1.0	0.3	0.7	0.5
Frequency of upper extremity static work					1.0	0.6	0.3
Frequency of using a handheld tool in a fixed position						1.0	0.8
Magnitude of hand-arm vibration (HAV)							1.0

Table S6. Biomechanical exposure and the incidence rate (IR) and relative risk (RR) for SIS surgery in workers still employed in construction trade at least one years within the 5-year period prior to follow up (N=125 123). RR were adjusted for BMI, smoking, age, and calendar time of surgery. The white-collar workers were used as reference.

	N	person-years	cases	IR	RR	95% CI
Frequency of working with hands above shoulder height						
reference	2 709	34 213	30	88	1.00	-
low	27 863	376 073	670	178	2.16	1.49-3.13
moderate	83 310	1 198 042	2 710	226	3.00	2.08-4.34
high	11 241	162 713	333	205	2.75	1.88-4.02
Magnitude of upper extremity loading						
reference	2 709	34 213	30	88	1.00	-
low	17 809	233 951	376	161	1.89	1.30-2.76
moderate	56 663	815 003	1 619	199	2.66	1.84-3.85
high	47 942	687 874	1718	250	3.29	2.28-4.76
Frequency of high grip force						
reference	2 709	34 213	30	88	1.00	-
low	19 715	259 959	427	164	1.94	1.33-2.82
moderate	43 748	633 450	1 246	197	2.67	1.84-3.86
high	58 951	843 419	2 040	242	3.18	2.21-4.60
Frequency of handheld tool use						
reference	2 709	34 213	30	88	1.00	-
low	20 932	277 022	475	172	2.03	1.40-2.95
moderate	9 558	135 955	285	210	2.70	1.84-3.96
high	91 924	1 323 851	2 953	223	2.98	2.07 -4.30
Frequency of using a handheld tool in a fixed position						
reference	2 709	34 213	30	88	1.00	-
low	41 087	564 189	1 086	193	2.40	1.66-3.48
moderate	5 259	75 418	159	211	2.74	1.84-4.07
high	76 068	1 097 221	2 468	225	2.98	2.06-4.29
Frequency of upper extremity static work						
reference	2 709	34 213	30	88	1.00	-
low	36 495	506 178	973	192	2.46	1.70-3.56
moderate	49 121	705 924	1 739	246	3.22	2.23-4.65
high	36 798	524 726	1 001	191	2.47	1.71-3.58
Magnitude of hand-arm vibration (HAV)						
reference	2 709	34 213	30	88	1.00	-
low	52 096	719 734	1 408	196	2.46	1.70-3.55
moderate	58 622	858 755	1 925	224	3.01	2.08-4.34
high	11 696	158 339	380	240	2.98	2.04-4.36

N – number workers; IR – incidence rate per 100 000 person-years; RR – relative risk; CI – confidence interval
